# Preoperative chemotherapy with a modified docetaxel, cisplatin, and S-1 regimen, followed by gastrectomy and lymphadenectomy for gastric cancer with bulky lymph nodes

**DOI:** 10.1007/s00595-025-03114-x

**Published:** 2025-08-25

**Authors:** Vo Duy Long, Dang Quang Thong, Tran Quang Dat, Doan Thuy Nguyen, Tran Vinh Tho, Tran Duy Phuoc, Nguyen Viet Hai, Nguyen Lam Vuong, Lam Quoc Trung, Nguyen Hoang Bac

**Affiliations:** 1 Department of Gastro-Intestinal Surgery, University Medical Center, University of Medicine and Pharmacy at Ho Chi Minh City, Ho Chi Minh City, Vietnam; 2 Department of General Surgery, Faculty of Medicine, University of Medicine and Pharmacy at Ho Chi Minh City, Ho Chi Minh City, Vietnam; 3 Department of Oncology, University Medical Center, University of Medicine and Pharmacy at Ho Chi Minh City, Ho Chi Minh City, Vietnam; 4 Department of Medical Statistics and Informatics, Faculty of Public Health, University of Medicine and Pharmacy at Ho Chi Minh City, Ho Chi Minh City, Vietnam; 5 University Medical Center at Ho Chi Minh City, 215 Hong Bang, Ward 11, District 5, Ho Chi Minh City, Vietnam

**Keywords:** Gastric cancer, Preoperative chemotherapy, Bulky lymph nodes, Clinical response rate, DCS regimen

## Abstract

**Purpose:**

The appropriate regimen and dosage of preoperative chemotherapy for gastric cancer (GC) with bulky lymph nodes (LNs) remain controversial. We conducted this study to evaluate the efficacy of preoperative chemotherapy using a modified regimen of docetaxel, cisplatin, and S-1 (DCS) for GC with bulky LNs, assessing feasibility, toxicity, response rate, and oncological outcomes.

**Methods:**

Thirty-two patients who had GC with bulky LNs diagnosed between Jan, 2018 and Oct, 2022 received three or four cycles of modified DCS regimen preoperatively. The primary outcome was 3 year overall survival (OS).

**Results:**

The completion rate of preoperative chemotherapy was 90.6% (4 cycles: 50.0%, 3 cycles: 40.6%). The disease control rate (DCR) and clinical response rate (RR) were 87.5% and 81.3%, respectively. Grade-3/4 neutropenia and anemia developed in 6.2% and 9.4%, respectively. Twenty-two patients with partial response (PR) agreed to undergo gastrectomy and LN dissection. Pathologic complete response (CR) was achieved in 15.6%. After surgery, there were no grade >  = 3 postoperative complications. The R0-resection rate was 65.6%. The 3 year OS and progression-free survival (PFS) rates were 43.0% and 37%, respectively, for all eligible patients. The 3 year OS and PFS of patients in the surgery group with negative para-aortic LNs were 58% and 47.0%, respectively.

**Conclusion:**

Preoperative chemotherapy with a modified DCS regimen demonstrated high tolerance, a clinical response rate, and satisfactory 3 year survival outcomes. Thus, a preoperative modified DCS regimen with 3–4 cycles is a promising approach for GC with bulky LNs.

## Introduction

Gastric cancer (GC) requires a multimodal approach, in which surgery remains the primary curative treatment for resectable tumors. However, curative surgery may not be achieved in patients with bulky lymph nodes (LNs) because of the direct invasion of bulky LNs into the major blood vessels and adjacent organs, resulting in R1/R2 resection [1, 2]. Thus, the optimal treatment strategy for GC with bulky LN involvement remains unclear.

It is thought that preoperative chemotherapy may improve the R0-resection rate and the prognosis of patients who have GC with bulky LNs [3–10]. Preoperative chemotherapy might help eradicate micro-metastasis, reduce the disease stage, and identify the drug sensitivity of the GC. Several studies have reported the efficacy of preoperative chemotherapy for patients with advanced GC including those with extensive LN metastasis [1, 2, 5, 11–15]. In Western countries, perioperative 5-FU, leucovorin, oxaliplatin, docetaxel (FLOT) or epirubicin, cisplatin, 5-FU (ECF), or epirubicin, cisplatin, and capecitabine (ECX) regimens have been recommended for advanced GC, based on the results of various prospective trials [16–18]. Conversely, the S-1-based regimens were found by several Asian studies to be favorable for advanced and/or GC with bulky LNs [4, 13, 19, 20]. The Japan Clinical Oncology Group (JCOG) 0405 trial of preoperative cisplatin and S-1 (CS) regimen in patients who had GC with bulky LNs and/or para-aortic lymph-node (PAN) metastasis, achieved a clinical response rate (RR) of 64.7% and a 3 year overall survival (OS) rate of 58.5%, with no treatment-related deaths [13]. Recently, several attempts have been made to apply triplet neoadjuvant regimens. In the JCOG1002 trial, the addition of docetaxel to the CS regimen (DCS) was expected to be a promising candidate for the next standard preoperative chemotherapy regimen for advanced GC [14, 21]. Despite achieving acceptable outcomes with an 85% R0-resection rate, preoperative chemotherapy with a traditional 2-cycle DCS regimen in this trial had low clinical RR (57.7%), with relatively high toxicity, and it did not improve long-term survival [15, 20]. Moreover, the JCOG1704 trial of docetaxel, oxaliplatin, and S-1 (DOS) regimen evoked the hypothesis that increasing the dose intensity of docetaxel and S-1 slightly would achieve a better response rate without increasing toxicity. The results demonstrated favorable pathological responses, but with a relatively high toxicity profile [15, 20]. Fushida proposed a modified-dosage docetaxel, cisplatin, S-1 (DCS) regimen by dividing the docetaxel dosage into biweekly schedules while maintaining the S-1 dose, to reduce toxicity and adverse effects and maintain the clinical response. However, the sample size was small and no survival outcomes were reported [15, 20]. Another recent randomized controlled trial (COMPASS-D) suggested that a 4-cycle DCS regimen might be better than a 2-cycle DCS, or a 2-cycle CS, or a 4-cycle CS regimen of neoadjuvant chemotherapy for advanced GC [22]. Thus, further investigations on preoperative chemotherapy for GC with bulky LNs using this modified DCS regimen with more cycles are warranted. We conducted this study to assess the effectiveness of preoperative chemotherapy using a dose-modified DCS regimen for the treatment of GC with bulky LNs, examining feasibility, toxicity, response rate, and oncological outcomes.

## Materials and methods

### Patients

**Fig. 1:**
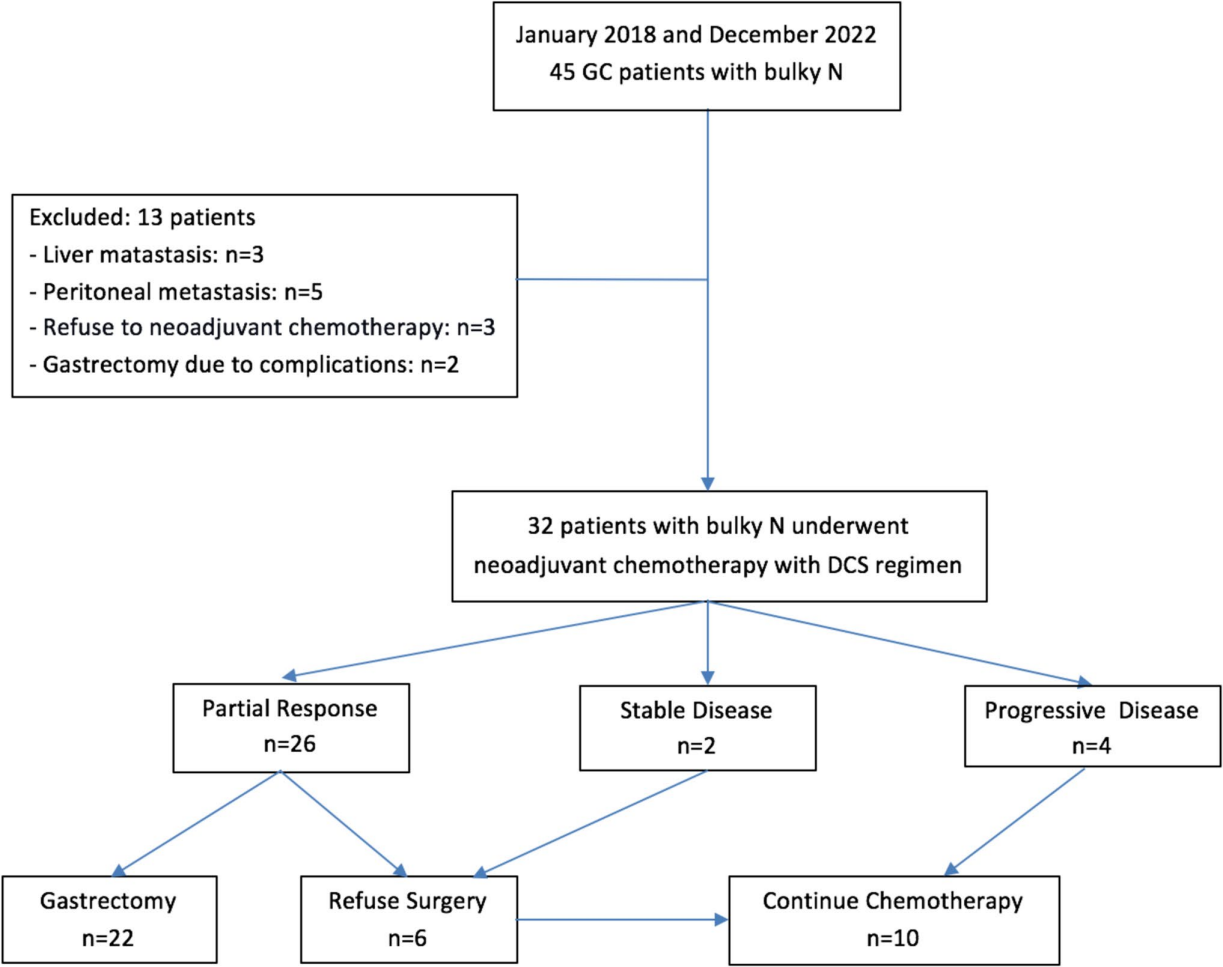
Flowchart of patient selection. *GC* Gastric Cancer, *Bulky LNs* Bulky Lymph Nodes, *DCS* Docetaxel, Cisplatin, and S-1

This was a retrospective cohort study of 32 Vietnamese patients with clinical bulky LN GC diagnosed between January, 2018 and October, 2022 at the Gastro-intestinal Surgical Department of the University Medical Center, a tertiary hospital in Ho Chi Minh City, Vietnam (Fig. 1). The study was approved by the Institutional Review Board of the hospital. Written informed consent was obtained from all patients before the collection of data.

The Japanese Gastric Cancer Association (JGCA) defines a bulky LN as a lymphatic nodal lesion surrounding the coeliac artery and its branches, with a diameter of > 3 cm or at least two adjacent nodes with a diameter of > 1.5 cm. GC with bulky LNs was diagnosed by a tumor board. Inclusion criteria comprised the following: (i) histologically confirmed gastric adenocarcinoma; (ii) clinical staging of bulky LNs; (iii) adequate hematological, liver, and renal function, including a white blood cell count between 4000 and 12,000/mm^3^, a neutrophil count ≥ 2,000/mm^3^, hemoglobin > 10 g/dL, a platelet count ≥ 100,000/mm^3^, aspartate aminotransferase (AST) and alanine aminotransferase (ALT) ≤ 100 IU/L, total bilirubin ≤ 1.5 mg/dL, creatinine ≤ 1.2 mg/dL, and creatinine clearance ≥ 60 mL/min; and (iv) an Eastern Cooperative Oncology Group (ECOG) performance status of 0 or 1. Exclusion criteria included distant organ metastasis (M1), except para-aortic LN (PAN) metastasis; concurrent cancer or a history of previous other cancers; prior chemotherapy; previous gastrectomy; central nervous system disorder; active hepatitis B; and pregnancy or breastfeeding.

### Preoperative chemotherapy and response assessment

Patients were administered three or four cycles of preoperative chemotherapy. From 2018 to 2020, the patients were scheduled to receive three cycles, but after the COMPASS-D trial in 2020 [22], we adjusted our regimen to include four cycles. Each cycle consisted of docetaxel (35 mg/m^2^, iv) and cisplatin (35 mg/m^2^, iv) on days 1 and 15, and S-1 (40 mg/m^2^, orally, twice daily) for 2 weeks from days 1 to 14, followed by a 2 week rest period. The total dosage per cycle was 70 mg/m^2^ docetaxel, 70 mg/m^2^ cisplatin, and 1120 mg/m^2^ S-1 (dose intensity of S-1 of 280 mg/m^2^/week). In the event of toxicity or adverse side effects, the subsequent cycle was delayed until recovery. Signs included a neutrophil count ≥ 1000/mm^3^, hemoglobin ≥ 10.0 g/dL, a platelet count ≥ 50,000/mm^3^, AST and ALT ≤ 150 IU/L, total bilirubin ≤ 2 mg/dL, and creatinine ≤ 1.2 mg/dL.

ECOG performance status was assessed, and complete blood cell counts, liver and renal function tests, and urinalysis were done routinely before each cycle to look for signs of toxicity and adverse events. At the end of the third and the fourth cycles, thoraco-abdominal computed tomography (CT) was performed to evaluate tumor response. We utilized the Response Evaluation Criteria in Solid Tumor (RECIST) 1.1 to assess the disease response [23]. The response categories were defined as follows: complete response (CR) denoted the complete disappearance of all target lesions, partial response (PR) indicated a reduction in the sum of the diameters of all target lesions by ≥ 30%, progressive disease (PD) was identified by an increase in the sum of the diameters of all target lesions by ≥ 20%, and stable disease (SD) was characterized by insufficient shrinkage to qualify for PR or insufficient increase to qualify for PD. Patients whose treatment achieved either CR or PR were regarded as having a clinical response. The disease control rate comprised the CR, PR, and SD rate. The Common Toxicity Criteria of the National Cancer Institute (NCI–CTC) 4.0 was used to report toxicities and adverse events [24].

### Surgery after preoperative chemotherapy

We performed gastrectomy with lymphadenectomy in accordance with the JGCA guidelines, within 2–4 weeks from the last day of preoperative chemotherapy. Based on the location of the primary tumor, we performed subtotal or total gastrectomy with lymphadenectomy via laparoscopy or laparotomy. Combined resection of the invaded organs was performed to achieve R0 resection. For patients with persistent PAN after preoperative chemotherapy, 16a2/b1 PAN dissection was carried out. Intraoperative lavage cytology was performed routinely, before and after gastrectomy. All surgical procedures were conducted by two experienced GC surgeons.

Curative resection (R0) was defined as the elimination of both macroscopic and microscopic diseases. R1 resection was defined as the macroscopic removal of the tumor, accompanied by microscopic evidence of residual tumor, as indicated by either a positive resection margin or preoperative lavage cytology (+).

### Adjuvant treatment

For patients who completed four cycles of preoperative chemotherapy, S-1 was administered for 1 year after surgery. For patients who completed three cycles preoperatively, a fourth cycle of DCS was administered, followed by S-1 for 1 year.

### Follow-up

The follow-up schedule followed the JGCA guidelines [25, 26]. Patients were followed up at 3 month intervals in the first 2 years, then at 6 month intervals for the next 3 years, and yearly thereafter. The follow-up visit consisted of physical examination, laboratory blood tests, and abdominal ultrasonography. Computed tomography was performed every 6 months for the first 3 years and then yearly. Endoscopy was performed every year. If a patient had symptoms or signs of recurrence or metastasis, CT and/or endoscopy were performed regardless of their follow-up schedule.

### Outcomes

The primary outcome was 3 year OS. The secondary outcomes included the rates of treatment-related death, tumor response, completion of preoperative chemotherapy, toxicity and adverse events, R0 resection, pathological complete response, short-term surgical outcomes, and 3 year progression-free survival (PFS). Long-term outcomes included OS and PFS for all the patients. OS was defined as the interval from the initiation of preoperative chemotherapy to death from any cause. PFS was defined as the time from the initiation of neoadjuvant chemotherapy to the first identification of disease progression or death from any cause. For patients who did not experience any of these events, OS and PFS were censored at the date of their last follow-up. The final follow-up date was June, 2025.

### Statistical analysis

Continuous variables are summarized as the mean ± standard deviation or median and interquartile range (IQR), and categorical variables are summarized as the number of patients and percentage. Baseline characteristics are summarized for all patients by three distinct groups: surgery, refusal of surgery, and PD. OS and PFS are summarized using the Kaplan–Meier method and visualized by the Kaplan–Meier curves. The Cox model was used to compare OS and PFS between groups. Results are reported as the hazard ratio (HR), 95% confidence interval (CI). The PFS was analyzed for patients with R0 resection only. All analyses were done using R statistical software version 4.1.3. Univariate analysis was performed using a two-sample *t* test for normally distributed numeric variables, the Wilcoxon rank-sum test for non-normally distributed numeric variables, and Fisher’s exact test for categorical variables. Multivariate analysis was conducted using logistic regression models with a stepwise backward procedure to identify independent risk factors of the OS rate.

## Results

### Patients’ characteristics

Table 1 summarizes the clinico-pathological characteristics of the patients. The mean age was 59.0 ± 10.5 years, with a male-to-female ratio of 5:3. Eighteen patients (56.2%) had cT4a disease, 13 (40.6%) had cT4b, and 9 (28.1%) had concurrent para aorta lymph-node involvement. Twelve patients (37.5%) with anemia at the time of admission received blood transfusions until the hemoglobin threshold had reached 10 g/dL.

**Table 1:** Patient characteristics

	All patients (*N* = 32)	Surgery (*N* = 22)	Declined surgery (*N* = 6)	PD (*N* = 4)	*p *value
Age (years)	59.0 ± 10.5	56.5 ± 10.0	64.7 ± 11.6	64.8 ± 8.5	0.081
Sex					> 0.999
Male	20 (62.5)	13 (59.1)	4 (66.7)	3 (75.0)	
Female	12 (37.5)	9 (40.9)	2 (33.3)	1 (25.0)	
BMI (kg/m2)	20.5 ± 2.6	20.8 ± 2.6	20.6 ± 1.9	18.8 ± 3.1	0.334
Nutritional status					0.179
Underweight (BMI < 18.5)	8 (25.0)	4 (18.2)	1 (16.7)	3 (75.0)	
Normal weight (BMI:18.5–24.9)	21 (65.6)	15 (68.2)	5 (83.3)	1 (25.0)	
Overweight (BMI:25–30)	3 (9.4)	3 (13.6)	0 (0.0)	0 (0.0)	
Obese (BMI > 30)	0 (0.0)	0 (0.0)	0 (0.0)	0 (0.0)	
Hypertension	7 (21.9)	6 (27.3)	1 (16.7)	0 (0.0)	0.812
Diabetes	0 (0.0)	0 (0.0)	0 (0.0)	0 (0.0)	
Cardiovascular disease	2 (6.2)	2 (9.1)	0 (0.0)	0 (0.0)	> 0.999
Chronic hepatic disease	3 (9.4)	1 (4.5)	2 (33.3)	0 (0.0)	0.117
Chronic lung disease	1 (3.1)	1 (4.5)	0 (0.0)	0 (0.0)	> 0.999
Previous stroke	2 (6.2)	2 (9.1)	0 (0.0)	0 (0.0)	> 0.999
Chronic renal disease	0 (0.0)	0 (0.0)	0 (0.0)	0 (0.0)	
History of laparotomy or laparoscopic surgery	1 (3.1)	0 (0.0)	1 (16.7)	0 (0.0)	0.313
CEA (U/L)	4.7 (1.9; 30.1)	3.9 (1.9; 10.8)	11.9 (2.1; 90.8)	134.1 (15.9; 257.1)	0.363
Preoperative WBC (g/L)	8.2 ± 2.3	8.2 ± 2.5	8.8 ± 1.9	7.7 ± 2.4	0.616
Hemoglobin (g/dL)	11.4 ± 2.5	11.3 ± 2.6	12.0 ± 2.9	11.1 ± 2.3	0.711
Anemia	12 (37.5)	9 (40.9)	1 (16.7)	2 (50.0)	0.536
Pre-CT tumor size (cm)	3.2 ± 2.0	3.2 ± 1.9	4.0 ± 2.6	2.0 ± 0.8	0.421
Differentiation status					0.652
Moderately differentiated	16 (50.0)	11 (50.0)	4 (66.7)	1 (25.0)	
Poorly differentiated	15 (46.9)	10 (45.5)	2 (33.3)	3 (75.0)	
Signet ring cell	1 (3.1)	1 (4.5)	0 (0.0)	0 (0.0)	
Para-aorta lymph node	9 (28.1)	5 (22.7)	1 (16.7)	3 (75.0)	0.113
Clinical T stage					**0.035**
T3	1 (3.1)	0 (0.0)	1 (16.7)	0 (0.0)	
T4a	18 (56.2)	10 (45.5)	5 (83.3)	3 (75.0)	
T4b	13 (40.6)	12 (54.5)	0 (0.0)	1 (25.0)	
Number of CT cycles					0.094
2	3 (9.4)	2 (9.1)	0 (0.0)	1 (25.0)	
3	13 (40.6)	8 (36.4)	3 (50.0)	2 (50.0)	
4	16 (50.0)	12 (54.5)	3 (50.0)	1 (25.0)	
Response after 4 cycles					** < 0.001**
CR	0 (0.0)	0 (0.0)	0 (0.0)	0 (0.0)	
PR	26 (81.3)	22 (100.0)	4 (66.7)	0 (0.0)	
SD	2 (6.2)	0 (0.0)	2 (33.3)	0 (0.0)	
PD	4 (12.5)	0 (0.0)	0 (0.0)	4 (100.0)	

Bold numbers indicates statistically significant differences between groups

Summary statistics are mean ± sd, n (%), and IQR

*BMI* body mass index, *CEA* carcinoembryonic antigen, *WBC* white blood cell, *CT* chemotherapy, *CR* complete response, *PR* partial response, *SD* stable disease, *PD* progress disease

### Tumor response

The total completion rate per protocol of three or four cycles was 90.6%. Sixteen patients (50.0%) received a complete course of four cycles and 13 (40.6%) received cycles of preoperative chemotherapy. The disease control rate was 87.5% (28 patients), including PR in 26 patients (81.3%) and SD in 2 patients (6.2%). There were no cases of CR. Four patients (12.5%) exhibited progressive disease (PD). Among them, three patients were found to have new metastatic sites (two in the liver and one in the peritoneum), while one patient showed progression in a target lesion (bulky LN). The clinical RR was 81.3% (all with PR). Among 28 patients for whom disease control was achieved, 22 of those with PR agreed to undergo gastrectomy and lymph-node dissection, while the remaining 6 (including 4 with PR and 2 with SD) declined surgery and continued chemotherapy. All four patients with PD received second-line chemotherapy (Table 1).

### Toxicity and adverse events

Table 2 shows the toxicity and adverse events. We observed three adverse events of grade 3–4, including neutropenia in 2 patients (6.2%) with grade 3, anemia in 3 patients (9.4%) with grade 3, and thrombocytopenia in 1 patient (3.1%) with grade 3. There was no treatment-related death.

**Table 2:** Treatment-related adverse events

	None	Grade 1	Grade 2	Grade 3	Grade 4
General fatigue	21 (65.6)	10 (31.2)	1 (3.1)	0 (0.0)	0 (0.0)
Vomiting	23 (71.9)	7 (21.9)	2 (6.2)	0 (0.0)	0 (0.0)
Diarrhea	23 (71.9)	7 (21.9)	2 (6.2)	0 (0.0)	0 (0.0)
Anorexia	30 (93.8)	2 (6.2)	0 (0.0)	0 (0.0)	0 (0.0)
Rash	31 (96.9)	1 (3.1)	0 (0.0)	0 (0.0)	0 (0.0)
Pneumonitis	32 (100.0)	0 (0.0)	0 (0.0)	0 (0.0)	0 (0.0)
Peripheral neuropathy	31 (96.9)	0 (0.0)	1 (3.1)	0 (0.0)	0 (0.0)
Stomatitis	29 (90.6)	3 (9.4)	0 (0.0)	0 (0.0)	0 (0.0)
Pigmentation	31 (96.9)	1 (3.1)	0 (0.0)	0 (0.0)	0 (0.0)
Leukopenia	20 (62.5)	8 (25.0)	4 (12.5)	0 (0.0)	0 (0.0)
Neutropenia	18 (56.2)	6 (18.8)	6 (18.8)	2 (6.2)	0 (0.0)
Febrile neutropenia	31 (96.9)	1 (3.1)	0 (0.0)	0 (0.0)	0 (0.0)
Anemia	15 (46.9)	10 (31.2)	4 (12.5)	3 (9.4)	0 (0.0)
Thrombocytopenia	30 (93.8)	1 (3.1)	0 (0.0)	1 (3.1)	0 (0.0)
Elevated bilirubin	28 (87.5)	4 (12.5)	0 (0.0)	0 (0.0)	0 (0.0)
Elevated SGOT	28 (87.5)	4 (12.5)	0 (0.0)	0 (0.0)	0 (0.0)
Elevated SGPT	28 (87.5)	4 (12.5)	0 (0.0)	0 (0.0)	0 (0.0)
Decreased BUN	32 (100.0)	0 (0.0)	0 (0.0)	0 (0.0)	0 (0.0)
Proteinuria	32 (100.0)	0 (0.0)	0 (0.0)	0 (0.0)	0 (0.0)
Hypokalemia	29 (93.5)	2 (6.5)	0 (0.0)	0 (0.0)	0 (0.0)
Hyponatremia	28 (90.3)	3 (9.7)	0 (0.0)	0 (0.0)	0 (0.0)

Summary statistics is *n* (%)

### Operative characteristics and short-term outcomes

Gastrectomy with lymphadenectomy was performed in 22 patients, as distal gastrectomy in 12 patients (54.5%) and as total gastrectomy in 10 patients (45.5%). Three patients underwent combined resection, as segmental transverse colectomy in one patient and as distal pancreato-splenectomy in two patients. D2 resection was performed in 15 patients (68.2%) and D2 + PAND was performed in 7 patients (31.8%). No patient had positive intraoperative lavage cytology.

The R0-resection rate in 32 eligible study patients was 65.6% (21/32). One patient had an R2 resection. The mean number of harvested lymph nodes was 20 (13;33). The overall complication rate in the 22 patients who underwent surgery was 22.7%, but there were no serious complications of Clavien–Dindo >  = 3 (Table 3).

**Table 3:** Operative characteristics

	*N*	Surgery (*N* = 22)
Operation type	22	
Laparoscopy		13 (59.1)
Open		9 (40.9)
Operative method	22	
Distal gastrectomy		12 (54.5)
Total gastrectomy		10 (45.5)
Borrmann	22	
1		1 (4.5)
2		10 (45.5)
3		10 (45.5)
4		1 (4.5)
Surgical tumor size (cm)	22	4 (3; 5)
Operating time (mins)	22	205 (180; 240)
Blood loss (ml)	22	100 (50; 138)
Combined surgery	22	3 (13.6)
Combined surgery specification	3	
Segmental transverse colectomy		1 (33.3)
Distal pancreato-splenectomy		2 (66.7)
Extent of lymph node dissection	22	
D2		15 (68.2)
D2 + PAND		7 (31.8)
Number of resected LNs	22	20 (13; 33)
Pathological T stage	22	
T0		5 (22.7)
T1		1 (4.5)
T2		1 (4.5)
T3		5 (22.7)
T4a		7 (31.8)
T4b		3 (13.6)
Pathological N stage	22	
N0		7 (31.8)
N1		4 (18.2)
N2		8 (36.4)
N3a		3 (13.6)
N3b		0 (0.0)
Curability	22	
R0		21 (95.5)
R1		0 (0.0)
R2		1 (4.5)
Adjuvant chemotherapy	22	
No		1 (4.5)
Not complete		4 (18.2)
Complete		17 (77.3)
Postoperative hospital stays (days)	22	8 (7; 9)
Time to flatus (days)	22	3 (3; 4)
Time to liquid diet (days)	22	3 (2; 4)
Anastomotic leakage	22	0 (0.0)
Anastomotic stricture	22	0 (0.0)
Duodenal stump leakage	22	0 (0.0)
Pancreatic fistula	22	1 (4.5)
Paralytic ileus	22	1 (4.5)
Bleeding	22	0 (0.0)
Intra-abdominal abscess	22	0 (0.0)
Wound infection	22	0 (0.0)
Early reoperation	22	0 (0.0)
Cardiovascular complications	22	0 (0.0)
Pneumonitis	22	2 (9.1)
Urinary retention	22	1 (4.5)
Clavien-Dindo classification	22	
1		2 (9.1)
2		3 (13.6)
> = 3		0 (0.0)

Summary statistics are *n* (%), mean ± sd, and IQR

A pathologic complete response (pCR) was achieved in 5 of the 32 eligible study patients (15.6%), while a complete pathological response only in the lymph nodes was seen in 2 patients.

### Long-term survival outcomes

**Fig. 2:**
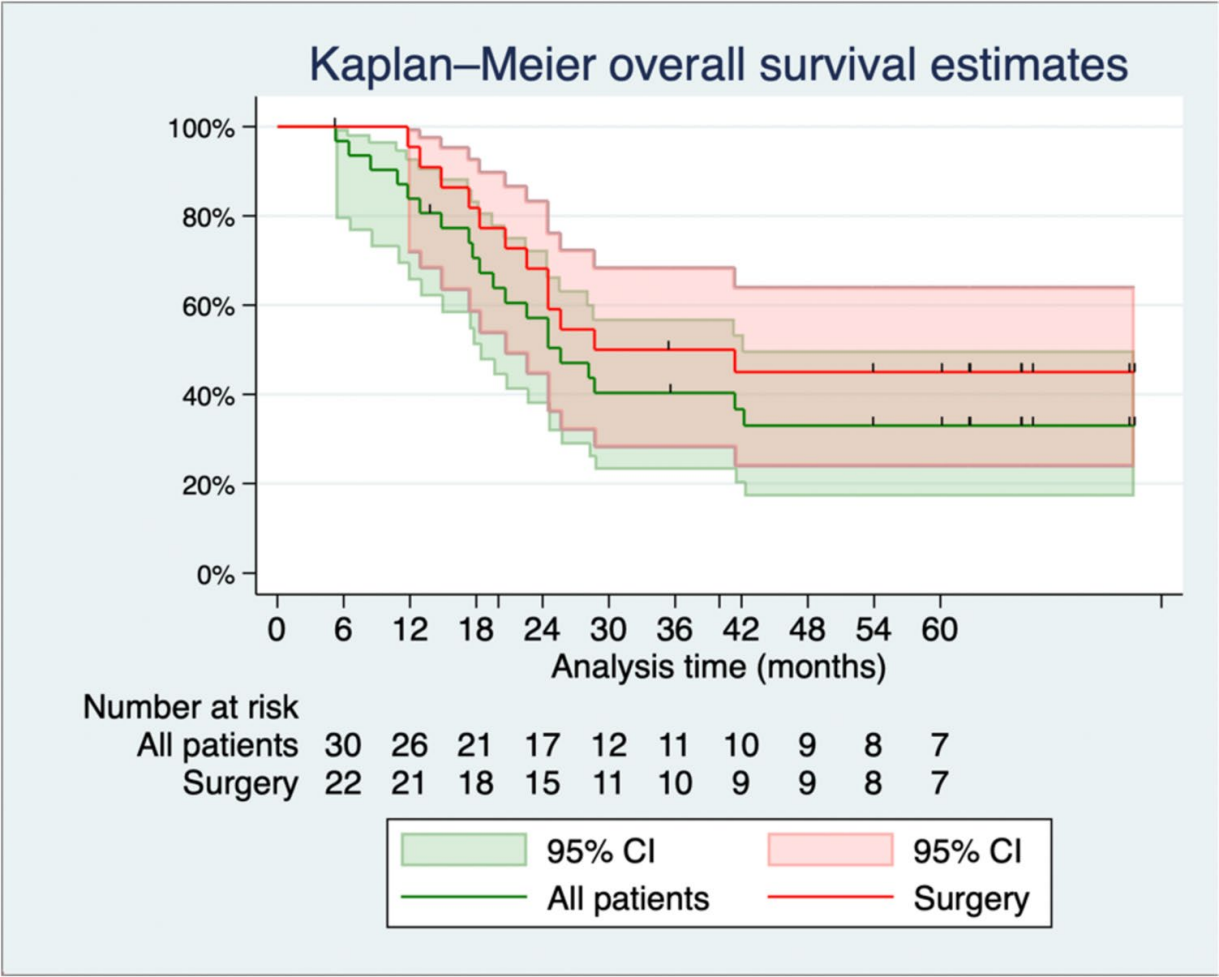
Kaplan–Meier curves for overall survival. *CI* Confidence Interval

The median length of follow-up was 24.5 (IQR: 17.7; 41.4) months in the total study population, and 32.0 (IQR: 22.4; 62.4) months in the surgery group. There were 22 deaths in the total population and 12 deaths in the surgery group. The median length of follow-up of the surviving patients was 64.8 (IQR: 55.7; 74.1) months. The 3 year OS and PFS rates were 43% and 37% for all eligible patients and 45% and 45% for the surgery group, respectively. For the subgroup of surgery patients with bulky LNs (+)/PAN (−), the 3 year OS and PFS rates were 58% and 47%, respectively (Fig. 2 and Table 4).

**Table 4:** Kaplan–Meier estimates of long-term survival

Kaplan–Meier probability (95% CI)			
	1 year	2 years	3 years
OS			
All eligible patients	84% (72%, 98%)	57% (38%, 72%)	43% (26%, 62%)
Surgery group	95% (87%, 100%)	50% (33%, 76%)	45% (28%, 72%)
Bulky LNs (+)/PAN (−)	94% (65%, 99%)	65% (38%, 82%)	58% (32%, 78%)
Refuse surgery	80% (52%, 100%)	27% (5.1%, 100%)	— (—, —)
PD	25% (4.6%, 100%)	— (—, —)	— (—, —)
PFS			
All eligible patients	74% (59%, 93%)	37% (22%, 60%)	37% (22%, 60%)
Surgery group	82% (67%, 100%)	45% (28%, 72%)	45% (28%, 72%)
Bulky LNs (+)/PAN (−)	76% (60%, 97%)	47% (30%, 74%)	47% (30%, 74%)

*CI* confidence interval, *OS* overall survival, Bulky LNs: bulky lymph node, *PAN* para-aortic lymph node,

*PD* progressive disease, *PFS* progression-free survival

Univariate analysis identified that factors including PAN (+) and incomplete adjuvant chemotherapy significantly reduced the overall survival rate of the surgery group. However, multivariate analysis did not identify any independent risk factors (Table 5).

**Table 5:** Univariable and multivariable analyses of factors associated with overall survival in the only surgery group

	Univariate model				Multivariate model		
Characteristic	*N*	HR	95% CI	*p *value	HR	95% CI	*p *value
Age (years)	22	1.07	0.99, 1.16	0.095			
Sex	22						
Male	13	1	Ref				
Female	9	0.61	0.16, 2.37	0.474			
BMI (kg/m2)	22	1.09	0.86, 1.37	0.464			
CEA (U/L)	22	0.99	0.96, 1.02	0.474			
Preoperative WBC (g/L)	22	1.15	0.91, 1.47	0.239			
Hemoglobin (g/dL)	22	1.04	0.81, 1.33	0.784			
Anemia	22	0.90	0.25, 3.19	0.868			
Pre-chemotherapy tumor size (cm)	22	0.96	0.67, 1.40	0.851			
Differentiation status	22						
Differentiated	11	1	Ref				
Undifferentiated	11	1.02	0.33, 3.16	0.976			
Para-aorta lymph node							
No	17	1	Ref				
Yes	5	4.72	1.15, 19.4	0.031	4.31	0.73, 25.41	0.107
Clinical T stage	22						
T4a	10	1	Ref				
T4b	12	1.04	0.33, 3.28	0.947			
Adjuvant chemotherapy	22						
Incomplete	5	1	Ref		1	Ref	
Complete	17	0.18	0.05, 0.67	0.013	0.18	0.02, 1.34	0.094
Number of preoperative CT cycles	22	1.08	0.41, 2.85	0.882			
Grade 3–4 adverse event							
No	17	1	Ref				
Yes	5	2.02	0.52, 7.86	0.313			
Operative method	22						
Distal gastrectomy	12	1	Ref				
Total gastrectomy	10	0.80	0.23, 2.86	0.736			

*HR* hazard ratio, *CI* confidence interval, *BMI* body mass index, *CEA* carcinoma embryonic antigen, *WBC* white blood cell, *CT* chemotherapy

### Recurrence pattern after surgery

Recurrence or metastasis developed in 11 patients (50%) from the surgery group, as hematogenous (2 patients), peritoneal (3 patients), distant LN (2 patients), and mixed type (4 patients).

## Discussion

GC patients with bulky LNs, considered to have borderline-resectable disease because of the technical challenges in *en-bloc* resection, a high risk of R1/R2 resection, and a risk of micro-metastasis, should be treated as a separate group to achieve better survival outcomes. In the present study, we expected to improve the long-term survival of our GC patients with bulky LNs through perioperative chemotherapy with a modified dosage of the DCS regimen followed by surgery. The findings demonstrated a high compliance rate, with more than three cycles of the preoperative DCS regimen achieved in 90.6%. Furthermore, the disease control rate was 87.5%, the clinical RR was 81.3%, and the R0-resection rate was 65.6%. Considering patients with PR and SD after preoperative chemotherapy, followed by gastrectomy, the 3 year OS reached 43%. These results advocate the combination of perioperative chemotherapy with the modified DCS regimen followed by surgery for this population, which until now has been supported by limited data [2, 7, 12–15, 20, 27].

The phase II JCOG0405 (CS regimen) [13] and JCOG1002 (DCS regimen) [15, 20] trials on patients with extended LN metastasis reported 5 year OS and PFS of around 53% and 48%, respectively. Although a confirmative comparison was not established, the JCOG investigators suggested that the DCS regimen did not improve short-term and long-term survival over the CS regimen. Furthermore, the JCOG1002 trial with a dose of docetaxel (40 mg/m^2^) and cisplatin (60 mg/m^2^) on day 1, resulted in high toxicity with rates of neutropenia, leukopenia, and febrile neutropenia of 39.6%, 18.9%, and 5.7%, respectively. Other studies also found that the rates of toxicity and adverse events were relatively high with these dosages [14, 15, 21, 28, 29]. A higher dose of docetaxel and cisplatin was thought to be related to a higher incidence of grade 3–4 hematological toxicity [30–32]. Moreover, in some studies, docetaxel-induced grade 3/4 neutropenia was observed more frequently in the Asian population than in non-Asian populations [6, 33]. Another argument from these studies relates to the length of preoperative chemotherapy; in particular, two cycles of preoperative DCS, as in the JCOG1002 trial, might not be optimal. The COMPASS-D trial suggested that a 4-cycle DCS regimen of neoadjuvant chemotherapy might be better than a 2-cycle DCS, a 2-cycle CS, or a 4-cycle CS regimen, for advanced GC [22].

From this perspective, we applied a more prolonged preoperative chemotherapy schedule in this study with three or four cycles of a modified-dose DCS regimen, as proposed by the phase II trial of Fushida and Tsutomu Hayashi [21, 22]. The total dose of docetaxel (70 mg/m^2^/cycle) and cisplatin (70 mg/m^2^/cycle), being higher than in other studies, was adjusted by dividing it into biweekly schedules to reduce toxicity-related adverse effects. The dose intensity of S-1 (280 mg/m^2^/week) remained the same as in the JCOG 1002 trial, but the dose intensity of docetaxel (17.5 mg/m^2^/week) and cisplatin (17.5 mg/m^2^/week) was higher than in other trials. Most of the toxicity and adverse events in our study were grade 1 or 2, with grade 3 neutropenia, thrombocytopenia, and anemia developing in 6.2%, 3.1%, and 9.4% of the patients, respectively. There were no grade 4 toxicities or adverse events. These results were remarkably lower than those reported in the JCOG 1407, JCOG 0405, and JCOG 1002 trials. Conversely, the disease control and clinical response rates were higher than in other studies [21, 22] (Table 6). Thus, a high completion and tolerance rate was achieved by this modified schedule in our study population.

**Table 6:** Summary of outcomes of the JCOG phase II trials and our results

Characteristic	Dose intensity	RR	R0	pCR	3-year OS	Neutropenia grade 3–4	Febrile neutropenia grade 3–4	Diarrhea grade 3–4	Anorexia grade 3–4
JCOG0001 (Irinotecan + Cisplatin)	NA	55	65	2 (1/55)	27	55	16	5	NA
JCOG0405 (Cisplatin + S-1)	C: 15 mg/m2/w S: 420 mg/m2/w x 3w, rest 1w	65	82	2 (1/51)	59	19	2	2	10
JCOG1002 (Docetaxel + Cisplatin + S-1)	D: 10 mg/m2/w C: 15 mg/m2/w S: 280 mg/m2/w x 2w, rest 2w	57.7	84.6	2 (1/52)	62.7	39.6	5.7	7.5	9.4
JCOG1704 (Docetaxel + Oxaliplatin + S-1)	D: 13.3 mg/m2/w O: 33.3 mg/m2/w S: 373 mg/m2/w x 2w, rest 1w	65	93	24 (11/46)	NA	24.4	8.9	8.9	15.6
Ours (Modified Docetaxel + Cisplatin + S-1)	D: 17.5 mg/m2/w C: 17.5 mg/m2/w biweekly (D1, D14) S: 280 mg/m2/w x 2w, rest 1w	81	65.6	15.6 (5/32)	43	6.2	0	0	0

*RR* response rate, *pCR* pathologic complete response, *OS* overall survival, NA not available, *C* Cisplatin, *D* Docetaxel, *S* S-1, *w* week, *D1* day 1, *D14* day 14

The OS rates in our study were lower than those reported by the JCOG0405 and JCOG1002 trials [13–15], being 43.0% at 3 years, partly because six patients for whom disease control was achieved refused surgery. Despite the higher response rate and comparable disease–control rate, the proportion of patients who proceeded to surgery following neoadjuvant chemotherapy was lower in our study (22/32 patients, 68.8%) than in the JCOG0405 study (49/52 patients, (94.2%), mostly because of the socioeconomic issues in our country. This lower surgical conversion rate may account for why OS in our study did not exceed that reported in the JCOG0405 trial.

Although our sample size was small, the survival results were comparable to the 3 year survival in the bulky LN (+)/PAN (-) subgroup in the above trials [13–15] and superior to the JCOG 0001 trial of the cisplatin–irinotecan regimen [2]. In the final aggregate, despite similar OS and PFS rates to those of the JCOG 0405 and 1002 trials, we expected that more patients in this population would benefit from the 3-/4-cycle modified-dose DCS regimen because of its lower toxicity, higher tolerance, and acceptable response rate. Moreover, four cycles of DCS with biweekly docetaxel and cisplatin may be more beneficial than two cycles, as demonstrated by the fact that all five cases (15.6%) of pathological complete response in our study were achieved by four cycles of DCS. Thus, we suggest three or four cycles of modified-dose DCS regimen as an acceptable choice for preoperative chemotherapy for GC patients with bulky LNs.

Five patients in our study suffered surgical complications, including pancreatic fistula, paralytic ileus, urinary retention, and pneumonitis. However, all these postoperative complications were minor; none of the patients experienced severe complications (ClavienDindo >  = 3). The feasibility and safety of extended gastrectomy after preoperative chemotherapy have been advocated, and our results are similar to those reported in the literature [10, 11, 14, 15, 20, 34, 35]. Overall, the rates of postoperative complications were comparable to those described previously for standard gastrectomy with D2 lymphadenectomy without preoperative chemotherapy. Moreover, there were no treatment-related deaths in our study. Therefore, extended gastrectomy after preoperative chemotherapy was deemed to be feasible and safe.

No prior studies have investigated the risk factors associated with survival. Our univariable analysis found that PAN (+) and incomplete adjuvant chemotherapy reduced the OS of the surgery group significantly. However, because of the small sample size, no independent risk factors were identified in the multivariable analyses.

It was unclear whether surgery is obligatory for patients who respond to chemotherapy, as there were no data on the survival of previous chemotherapy-only groups. Our findings demonstrated that patients with either PR or SD after preoperative chemotherapy benefited from perioperative chemotherapy combined with gastrectomy over chemotherapy alone, with significant differences in 1-, 3- and 5 year OS and PFS rates. Thus, we strongly recommended gastrectomy after preoperative chemotherapy over chemotherapy alone for patients with a response or SD after preoperative DCS chemotherapy.

This study had some limitations. First, none of the patients underwent positron emission topography or diagnostic laparoscopy before neoadjuvant chemotherapy was initiated, which might have led to the underdiagnosis of occult metastases. Second, the sample size of our study was small. Third, by the time of analysis in June 2025, the median follow-up period was only 24.5 (IQR: 17.7; 41.4) months. All patients had reached 3 years of follow-up (10 surviving patients) or the 3 year endpoint (22 deaths). Therefore, estimating the 5 year survival rate was premature at this time, although it is important to highlight that GC with bulky LNs is uncommon, making large-scale studies focusing on this cohort challenging in terms of recruitment. The strength of our study lies in its additional contribution to the existing literature by showing the potential benefits of a dose-modified DCS regimen for improving tolerability and reducing toxicity while maintaining favorable oncological outcomes, such as high response, enabling R0 resection, and substantial survival rates. Further prospective, multi-center studies with larger cohorts are warranted to validate these findings.

In conclusion, preoperative chemotherapy with a modified DCS regimen followed by gastrectomy and lymphadenectomy demonstrated a high tolerance, tumor response rate, and satisfactory 3 year survival outcomes for GC patients with bulky LNs. Thus, three-to-four cycles of the modified-dose DCS regimen preoperatively is a promising approach for GC with bulky LNs.

## Data Availability

The data presented in this study are available on request from the corresponding author.
